# Systematic Meta-analysis Revealed an Association of PGC-1*α* rs8192678 Polymorphism in Type 2 Diabetes Mellitus

**DOI:** 10.1155/2019/2970401

**Published:** 2019-03-03

**Authors:** Wanning Xia, Nanxing Chen, Wenjia Peng, Xianjie Jia, Ying Yu, Xuesen Wu, Huaiquan Gao

**Affiliations:** ^1^School of Public Health, Bengbu Medical University, Bengbu, Anhui 233030, China; ^2^Department of Physiology, Bengbu Medical University, Bengbu, Anhui 233030, China

## Abstract

**Background:**

Genome-wide association study (GWAS) provides an unprecedented opportunity to reveal substantial genetic contribution to type 2 diabetes mellitus (T2DM) and glycemic identification of allelic heterogeneity and population-specific genetic variants, yet it also faces difficulty due to the vast amount of potential confounding factors and limited availability of clinical data. To identify responsible susceptibility loci and genomic polymorphism for T2DM and glycemic traits, we have systematically investigated a genome-wide association study related to T2DM. Although GWAS has captured many common genetic variations, which are related to T2DM, each risk allele (RA) of single-nucleotide polymorphisms (SNPs) at these loci is not conclusive. Therefore, it is common to present a combination of several SNPs to infer T2DM risk, yet it is still insufficient to be deterministic. To streamline the identification of a deterministic genetic variation in T2DM, we developed this meta-analysis as a showcase to comprehensively identify the association between cumulative RAs and T2DM risk by combining different studies in reported literature and databases. After all, we identified that PGC-1*α* rs8192678 polymorphism can be considered as a potentially deterministic biomarker in T2DM risk. Previous studies have potentially linked PGC-1*α* rs8192678 polymorphism to type 2 diabetes mellitus (T2DM) risk, but the results remain inconsistent in different populations and are not conclusive. We developed a new meta-analysis approach to systematically identify the association between PGC-1*α* rs8192678 polymorphism and T2DM, and we have comprehensively assessed different ethnic groups to validate our findings.

**Methods:**

We performed comprehensive information retrieval and knowledge discovery meta-analysis by searching extensively published literature and different electronic databases to acquire eligible studies for the above association study. We developed a method to use pooled odds ratios (ORs) and their corresponding 95% confidence intervals (CIs) in five genetic models (allelic, dominant, recessive, homozygous, and heterozygous genetic models) to identify the relationship among ethnicity subgroup analyses comprehensively.

**Results:**

We identified 20 eligible studies consisting of 16,182 subjects (8,038 cases and 8,144 controls) in our meta-analysis. PGC-1*α* rs8192678 polymorphisms of all subjects showed a significant association with T2DM susceptibility under all genetic models: allelic (OR: 1.24, 95% CI: 1.13-1.35), dominant (OR: 1.27, 95% CI: 1.14-1.42), recessive (OR: 1.24, 95% CI: 1.14-1.36), homozygous (OR: 1.40, 95% CI: 1.20-1.64), and heterozygous (OR: 1.20, 95% CI: 1.06-1.35). In the subgroup analysis, we identified a significant association between PGC-1*α* rs8192678 polymorphism and T2DM in the Caucasian and Indian populations under all genetic models we investigated. This is the most comprehensive study of the subject to date.

**Conclusion:**

Our development of meta-analysis revealed that the minor allele (A) carriers, especially AA genotype carriers, can lead to risk of T2DM in the Caucasian and Indian populations. This is the first report that such risk has been confirmed. Our finding shed new light into the genetic alteration in T2DM.

## 1. Introduction

T2DM is a significant global health issue with potential life-threatening complications if not controlled well. Over 451 million people worldwide were estimated to live with diabetes according to the International Diabetes Federation [1]. T2DM continues to rise worldwide and is projected to rise to 693 million by 2045. T2DM is the most prevalent type of diabetes, accounting for around 90% of all types of diabetes. T2DM is characterized by hyperglycemia caused by impaired insulin secretion and insulin resistance. Hyperglycemia, if not controlled well, can eventually cause severe damages to various human organs, leading to serious complications, such as cardiovascular dysfunction, neuropathological damages, kidney failure, retinopathological changes, and even blindness. Although the etiology for diabetes remains unclear, both environmental and genetic factors are known to be related to the development of T2DM [2, 3]. A large number of susceptibility genes have been reported via a genome-wide association study (GWAS). Due to differences in race and region, most susceptible genes only have a weak effect on the risk of T2DM and have not been replicated by a large population.

After comprehensive investigations on T2DM, we found that peroxisome proliferator-activated receptor *γ* coactivator-1*α* (PGC-1*α*) is crucially important to T2DM. PGC-1*α* is a multifunctional regulatory factor originally identified as a coactivator of PPAR*γ* in 1998 [4]. PGC-1*α*, located on chromosome 4p15.1, is highly expressed in the liver and skeletal muscle and is involved in maintaining glucose, lipid, and energy homeostasis. PGC-1*α*, as a crucial gene regulatory element in various metabolic processes, has been shown to play pivotal roles in the development of obesity, insulin resistance, and T2DM [5–7]. In the previous studies from different countries, the association of several single-nucleotide polymorphisms (SNPs) of PGC-1*α*, such as rs8192678G>A, rs2970847C>T, rs3736265G>A, and rs3755863 C>T, with risk of T2DM were studied [8–11], while an A allele (mutant type) of the rs8192678 G>A polymorphism in PGC-1*α* gene has been speculated in T2DM risk in some studies [12–14]; such possible link was not identified from other studies [15]. This discrepancy may be due to the relatively small sample size and differences among ethnicities. It is important that we can develop a meta-analysis approach to overcome limitations and disadvantages of previous studies by combining various studies and including ethnicity for reaching a conclusive validation.

In this study, the association of PGC-1*α* rs8192678 (G>A) polymorphism and T2DM was extensively investigated more comprehensively by combining relevant studies. In addition, we combined ethnicity in all eligible studies to elucidate the explicit association of PGC-1*α* rs8192678 (G>A) polymorphism and T2DM.

## 2. Methods

### 2.1. Strategy of Literature Search and Database Utilization

The electronic databases, both in English and Chinese, including PubMed, Springer, Chinese National Knowledge Infrastructure, SinoMed, and Wanfang were searched for collecting relevant literatures published from January 2001 to May 2018. For all databases, we expanded broad search terms such as “PPARGC1A,” “PGC-1alpha”, “T2DM,” “diabetic,” “insulin,” “hyperglycemia,” “polymorphism,” and “mutation” for more comprehensive information harvest. We do not set up any other filter to ensure all relevant information will be included in our investigations.

#### 2.1.1. Selection Criteria

To ensure the comprehensiveness and integration of information retrieval, we imposed the following criteria for all relevant literatures:

1. Studies published in peer-reviewed journals
2. Case-control studies referring to the association between PGC-1*α* polymorphism and T2DM risk
3. Cases and controls with allele and genotype frequencies
4. Genetic variants of controls that met the Hardy-Weinberg equilibrium (HWE, *P* > 0.05).

The following studies were excluded:

1. Studies without sufficient data
2. Non-case-control studies
3. Studies based on family
4. Redundant studies of duplicated data

### 2.2. Data Extraction

Two researchers (Wan-ning Xia, Nan-xing Chen) independently reviewed full texts to carefully select eligible papers and extracted all information, including the first author, year of publication, ethnicity of subjects, age, gender, BMI, diagnostic criteria, sample size, molecular methods for genotype testing, and allele and genotype distribution in cases and controls. The third researcher (Wen-jia Peng) reviewed, cross-verified, and then finalized the carefully selected literature. Any disagreements were resolved by all 3 researchers through careful reexaminations altogether. If similar data were reported more than two times in different papers, only one was adopted in order to eliminate any bias. The genotype frequencies of controls in all included literatures were verified by HWE.

### 2.3. Quality Assessment

The Newcastle-Ottawa Scale (NOS) [16] tool was used to appraise the quality of studies by at least two researchers independently to cross-validate the result. This scale consists of three categories (Selection, Comparability, and Exposure) with eight items in total. A study can be awarded a maximum of one star for each numbered item within the Selection and Exposure categories. A maximum of two stars can be given for Comparability. Item scores range from 0 to 9 stars, and an overall score of more than 6 stars was regarded as high quality [17] .

### 2.4. Statistical Analyses

All statistical analyses were performed using STATA version 12.0 and RevMan v5.3 (the Cochrane Collaboration) software. The G allele is wild, and the A allele is mutated for the rs8192678 G>A polymorphism in the PGC-1*α* gene. The pooled odds ratio (OR) with a 95% confidence interval (95% CI) was calculated in different genetic models, allelic (A vs. G), dominant (AG + AA vs. GG), recessive (AA vs. AG + GG), homozygous (AA vs. GG), and heterozygous (AG vs. GG) genetic models. Subgroup analysis by ethnicity was also conducted. Heterogeneity across studies was assessed via a chi-square test and *I*^2^. The fixed effects model was used for no heterogeneity (*I*^2^ < 50%, *P* > 0.05), whereas the random effects model was selected. The HWE was examined via the chi-square test. A funnel plot was used to detect potential publication bias, and the funnel plot symmetry was evaluated using Egger's linear regression testing on the OR. If publication bias was indicated, we further evaluated the number of missing studies by the Trim and Fill method and recalculated the pooled risk estimation with the addition of those missing studies [18].

## 3. Results

### 3.1. Characteristics of the Studies

**Figure 1:**
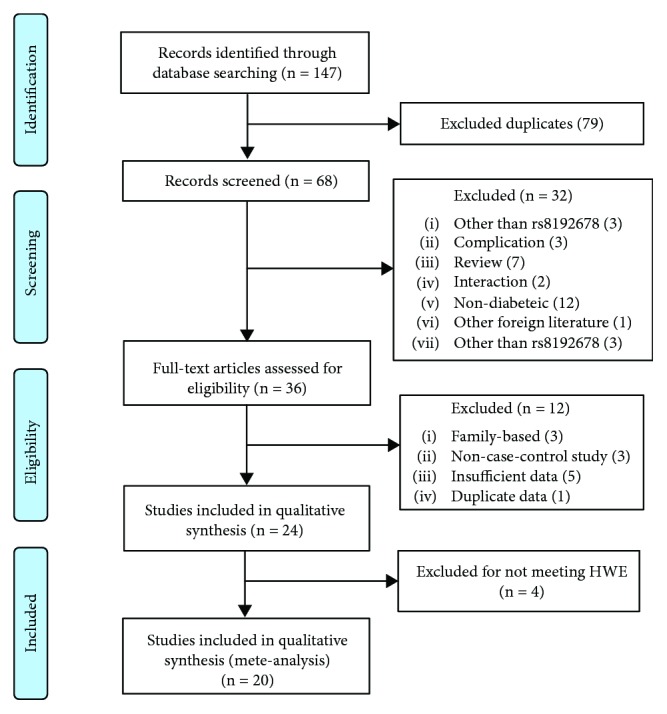
The flow chart of literature search and selection.

After duplicates were removed, 68 papers of potentially relevant studies were included initially. Then, 32 papers were excluded by reading abstracts carefully. Among those remaining relevant studies, 16 papers (3 studies based on family, 3 non-case-control studies, 5 studies with insufficient data, 1 duplicate data study, and 4 studies not meting HWE) were also excluded after reading the full texts (Figure 1). Thus, 20 papers consisting of 16,182 subjects (8,038 cases and 8,144 controls) were finally included in this meta-analysis. The characteristics of included studies are listed in Table 1. 10 studies were undertaken in an East Asian population, 7 studies were in a Caucasian population, 2 studies were in an Indian population, and 1 study was in African people. Quality scores ranged from 4 to 8 stars with a median value of 6.48 (Table 2).

### 3.2. Meta-analysis Results

**Figure 2:**
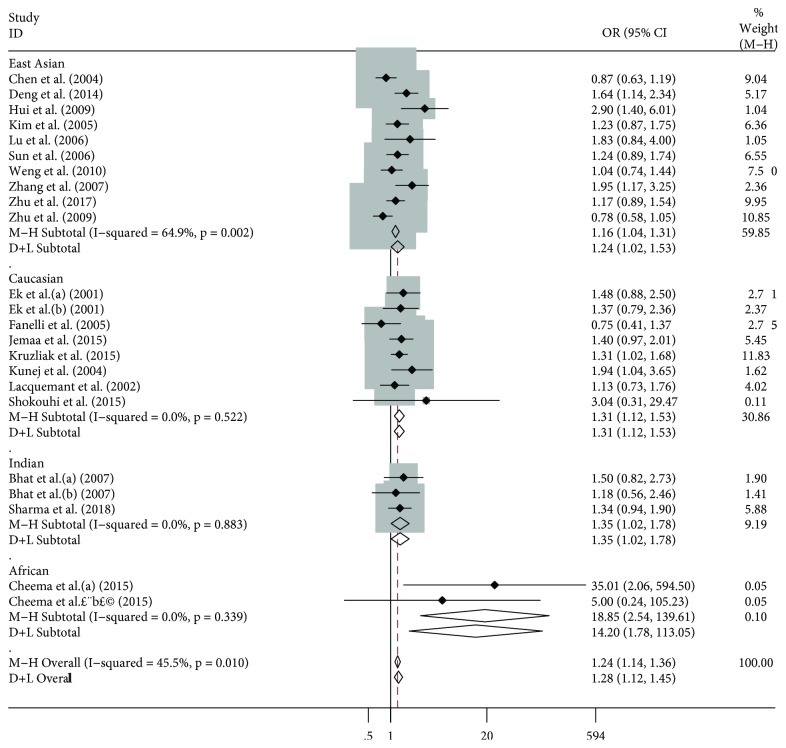
Forest plot of T2DM associated with PGC-1*α* rs8192678 polymorphism under recessive genetic model (AA vs. AG + GG).

For the study of the whole population included in our investigation, PGC-1*α* rs8192678 polymorphisms have shown a significant association with T2DM risk under allelic (OR: 1.24, 95% CI: 1.13-1.35), dominant (OR: 1.27, 95% CI: 1.14-1.42), recessive (OR: 1.24, 95% CI: 1.14-1.36), homozygous (OR: 1.40, 95% CI: 1.20-1.64), and heterozygous (OR: 1.20, 95% CI: 1.06-1.35) genetic models (Table 3). A Fixed Effect model was only used to the recessive genetic model (Figure 2).

In a subgroup analysis, a significant association between PGC-1*α* rs8192678 polymorphisms and T2DM was only found in the East Asian population under allelic (OR: 1.15, 95% CI: 1.02-1.29), recessive (OR: 1.17, 95% CI: 1.04-1.31), and homozygous (OR: 1.31, 95% CI: 1.04-1.64) genetic models (Table 3).

A significant association was observed in the Caucasian population under allelic (OR: 1.28, 95% CI: 1.09-1.49), dominant (OR: 1.37, 95% CI: 1.10-1.71), recessive (OR: 1.31, 95% CI: 1.12-1.53), homozygous (OR: 1.47, 95% CI: 1.21-1.79), and heterozygous (OR: 1.32, 95% CI: 1.05-1.66) genetic models (Table 3).

This significant association was also found in the Indian population under allelic (OR: 1.35, 95% CI: 1.12-1.62), dominant (OR: 1.54, 95% CI: 1.12-2.11), recessive (OR: 1.35, 95% CI: 1.02-1.78), homozygous (OR: 1.59, 95% CI: 1.18-2.14), and heterozygous (OR: 1.52, 95% CI: 1.08-2.13) genetic models (Table 3).

No AA genotype carrier was found in the African population, nor was there any significant association detected in this study.

### 3.3. Publication Bias

**Figure 3:**
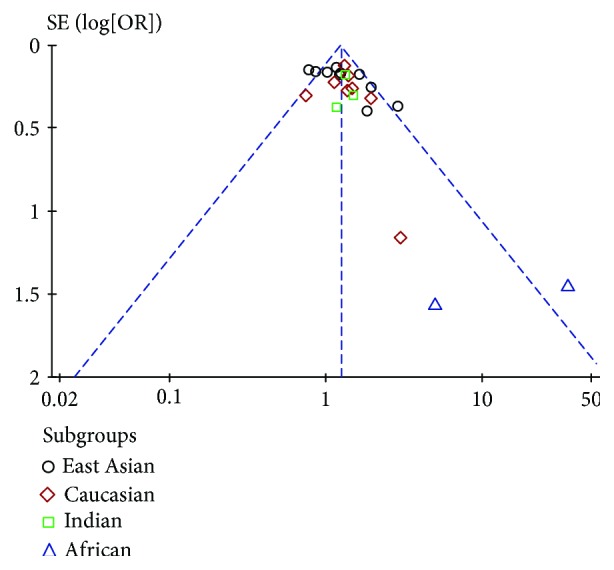
Funnel plot of T2DM associated with PGC-1*α* rs8192678 polymorphism under recessive genetic model (AA vs. AG + GG).

**Figure 4:**
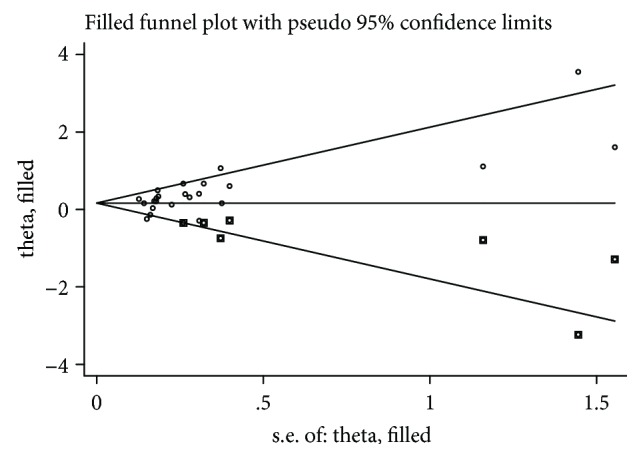
Filled funnel plot of T2DM associated with PGC-1*α* rs8192678 polymorphism under recessive genetic model (AA vs. AG + GG).

The publication bias of these studies was assessed under a recessive genetic model by using the funnel plot (Figure 3). Egger's linear regression test indicated possible publication bias for the association (*T* = 3.22, *P* = 0.004) under the recessive genetic model. The Trim and Fill method was used to recalculate the pooled risk estimate. There was some indication of asymmetry (seven studies trimmed), but the results were still stable before and after this analysis (Figure 4).

### 3.4. Sensitivity Analysis

**Figure 5:**
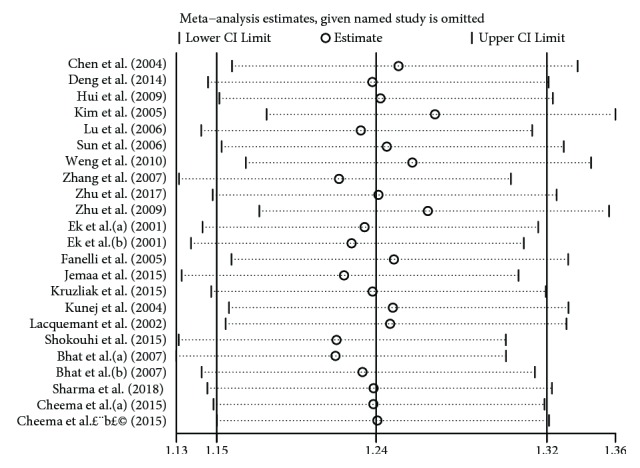
Sensitivity analysis under recessive genetic model (AA vs. AG + GG).

We used the leave-one-out method in the sensitivity analysis, whereas only one article was excluded each time. The removal of any one study from this meta-analysis did not change the association between PGC-1*α* rs8192678 polymorphisms and T2DM susceptibility under the recessive genetic model. This suggests that our results are stable, reliable, and robust (Figure 5).

## 4. Discussion

Many epidemiological studies have investigated the genetic risk of developing T2DM using multiple single-nucleotide polymorphism- (SNP-) based GWAS approaches. However, up to today, the quantitative association of cumulative risk alleles (RAs) of such SNPs with T2DM risk has not been deterministic, limiting the potential applications of this type of research. Hence, the aim of this study was to identify deterministic genetic alteration associated with T2DM. The comprehensive meta-analysis of cross-ethnicity studies revealed T2DM risk in relation to PGC-1*α* gene rs8192678 polymorphism. We firstly report this current meta-analysis that carrying one RA in T2DM-associated SNPs can be associated with a clear risk of prevalent or incident T2DM, and we have validated our finding statistically.

In this meta-analysis, the association between PGC-1*α* gene rs8192678 polymorphism and T2DM susceptibility was detected under different genetic models. Individuals with A allele carriers can have an increased risk of T2DM, especially among the Caucasian and Indian populations. After subgroup analysis, a significant association was detected in the Caucasian population under all genetic models, and also found in the Indian population. Furthermore, people with AA genotype carriers can have a higher risk of T2DM compared to people with other genotype carriers. The association was detected in the East Asian population under recessive and homozygous genetic models. No significant association was found among the Africans because of the only one study with small sample size and without AA genotype carriers in control. Heterogeneity showed reduction after ethnicity subgroup analysis. This infers that ethnicity can be a main reason for heterogeneity. The possible publication bias, as detected by Funnel plot and Egger's linear regression test, suggests that further studies are still needed to validate the conclusions, while the sensitivity analysis supports that our results are stable, robust, and reliable.

Initially, Ek et al. [19] performed a case-control study about the relationship between PGC-1*α* rs8192678 polymorphism and T2DM. The association between PGC-1*α* polymorphism and T2DM, insulin secretion, and other related indicators was also explored in different countries, but with inconsistent and contradictory conclusions [20–23]. The discrepancy among original studies was common in any study of complex disease. The reasons for the discrepancy can be multifaceted, including sample size, ethnicity differences, study design, and inclusion criteria. In 2006, Barroso et al. [24] found a significant association between PGC-1*α* rs8192678 polymorphism and T2DM in the Caucasian population. Eight Caucasian studies from 2001 to 2005 were included in their meta-analysis, and only 2 genetic models (allelic, additive) were adopted. Because both unrelated and genealogical samples were included in their analysis, the comparability of studies might be decreased and their conclusion may be more limited, compared to the results from our study. In 2011, Yang et al. [10] also performed a similar meta-analysis, including studies from 2001 to 2010, and found an obvious association among Indians but not in Caucasians. However, only an allelic genetic model was adopted with subgroup analysis stratified by ethnicity. The studies included in the meta-analysis comprised family-based studies, redundantly repeated data studies, and studies that are not matched in HWE. Sharma et al. [25] investigated the possible association between PGC-1*α* rs8192678 polymorphism and T2DM but only included 8 studies. However, a high level of heterogeneity among these studies was identified with an *I*^2^ value of 81.58%. Hence, the reliability of the previous results has been questionable. We performed a much more comprehensive investigation, and in our meta-analysis, all included studies were detected by HWE. The selection of literature by inclusion and exclusion criteria was strictly followed to reduce the potential selection bias. We performed more comprehensively five genetic models with subgroup analysis stratified by ethnicity and literatures (after 2010). Therefore, our much more comprehensive approach offers more reliable results with new updates.

Despite our promising results, several potential limitations should also be addressed. Firstly, T2DM is a complex multifactorial disease, and we only considered the individual polymorphism without taking into account the interaction with other polymorphisms or environmental factors (dietary pattern, lifestyle, behavioral habits, etc.). Secondly, the study did not consider other relevant factors (gender, age, etc.), due to the limitation of availability of original research data. Thirdly, obesity is an important intermediate factor in the development of T2DM, but the definitions of obesity were different or not available in our included studies. It would be valuable and interesting to include obesity in the association with subgroup analysis. Finally, sample sizes were insufficient in the Indian and African populations. However, even though those factors are obviously related, none of them is indispensible in T2DM development, since T2DM can occur without any of the above factors. Therefore, despite of those limitations, a reliable conclusion can be still made from our comprehensive study.

## 5. Conclusion

A significant association between PGC-1*α* rs8192678 polymorphism and T2DM susceptibility has been detected in this meta-analysis. Individuals with A allele, especially AA genotype carriers, can be more susceptible to T2DM in the Caucasian and Indian populations. Such association in East Asian and African populations needs to be further explored in larger sample studies. Further research about the interaction effect on PGC-1*α* rs8192678 polymorphism with other SNPs or environmental factors will be followed. Our finding revealed an explicit genetic variation in T2DM development. Our study provides further insight into the mechanisms by which genetic variation influences type 2 diabetes risk and glycemic traits, and it further supports that the genetic variant for type 2 diabetes risk can confer the risk diversity among different ethnicities.

## Figures and Tables

**Table 1:** Characteristics of the included studies about the association between PGC-1*α*rs8192678 polymorphism and T2DM.

Author	Year	Ethnicity	T2DM				Control				Matching variables	Chi-square values of HWE	*P* value
			GG	GA	AA	Total	GG	GA	AA	Total			
Chen et al. [26]	2004	Chinese	155	255	84	494	185	264	106	555	Age, gender	0.466	0.495
De-yao et al. [27]	2014	Chinese	165	175	90	430	190	181	60	431	Age, gender	2.492	0.115
Hui et al. [28]	2009	Chinese	71	28	41	140	47	30	11	88	Gender, BMI	1.276	0.259
Kim et al. [14]	2005	Korean	251	355	152	762	88	163	51	303	Gender	2.779	0.096
Lu et al. [29]	2006	Chinese	44	55	21	120	54	41	11	106	—	0.581	0.446
Sun et al. [30]	2006	Chinese	122	190	78	390	181	256	88	525	Age, gender, BMI	0.025	0.876
Weng et al. [13]	2010	Chinese	91	129	56	276	340	502	207	1049	Age, gender, BMI	0.78	0.377
Zhang et al. [31]	2007	Chinese	97	121	45	263	144	111	27	282	Age, BMI	0.679	0.41
Zhu et al. [8]	2017	Chinese	138	251	108	502	250	382	150	784	Age, gender	0.036	0.85
Zhu et al. [32]	2009	Chinese	181	303	111	595	143	240	112	495	Gender, BMI	0.347	0.556
Ek et al. (a) [19]	2001	Caucasian	186	200	68	454	97	80	21	198	—	0.541	0.462
Ek et al. (b) [19]	2001	Caucasian	76	97	28	201	146	116	31	293	—	1.2	0.273
Fanelli et al. [33]	2005	Caucasian	51	56	18	125	86	96	41	223	Gender	2.431	0.126
Jemaa et al. [34]	2015	Caucasian	166	231	90	478	176	170	56	402	Age, gender	2.055	0.152
Kruzliak et al. [12]	2015	Caucasian	80	334	467	881	40	147	161	348	Age, gender	0.529	0.467
Kunej et al. [35]	2004	Caucasian	141	129	35	305	114	111	15	240	Age, gender, BMI	3.156	0.076
Lacquemant et al. [15]	2002	Caucasian	129	137	44	310	163	159	47	369	—	0.705	0.401
Shokouhi et al. [9]	2015	Caucasian	127	43	3	173	159	13	1	173	Age	1.531	0.216
Bhat et al. (a) [11]	2007	Indian	68	103	28	199	112	80	21	213	BMI	1.401	0.237
Bhat et al. (b) [11]	2007	Indian	69	70	13	152	143	96	19	258	BMI	0.269	0.604
Sharma et al. [25]	2018	Indian	220	254	80	554	258	249	64	571	Age, sex	0.112	0.737
Cheema et al. (a) [36]	2015	African	92	4	14	110	102	14	0	116	Gender, BMI	0.478	0.489
Cheema et al. (b) [36]	2015	African	103	19	2	124	104	18	0	122	Gender	0.774	0.379

HWE: Hardy-Weinberg equilibrium; *P* value: *P* value of HWE.

**Table 2:** Results of quality assessment by NOS.

Study ID	Year	Selection				Comparability	Exposure			Score
		Adequate definition of cases	Representativeness of cases	Selection of controls	Definition of controls	Control for important factors	Ascertainment of exposure	Same method of ascertainment for cases and controls	Non-response rate	
Bhat et al. (a)	2007	☆	☆	—	☆	☆	☆	☆	☆	7
Bhat et al. (b)	2007	☆	☆	—	☆	☆	☆	☆	☆	7
Cheema et al. (a)	2015	☆	☆	—	☆	☆	☆	☆	☆	7
Cheema et al. (b)	2015	☆	☆	—	☆	—	☆	☆	☆	6
Chen et al.	2004	☆	☆	☆	☆	☆	☆	☆	☆	8
De-yao et al.	2014	—	☆	☆	☆	☆	☆	☆	☆	7
Ek et al. (a)	2001	☆	—	☆	☆	—	☆	☆	☆	6
Ek et al. (b)	2001	☆	—	☆	☆	—	☆	☆	☆	6
Fanelli et al.	2005	☆	☆	—	☆	—	—	☆	—	4
Hui et al.	2009	—	☆	—	☆	☆	☆	☆	☆	6
Jemaa et al.	2015	—	☆	☆	☆	☆	☆	☆	☆	7
Kim et al.	2005	☆	☆	—	☆	—	☆	☆	☆	6
Kruzliak et al.	2015	—	☆	☆	☆	☆	☆	☆	☆	7
Kunej et al.	2004	—	☆	—	—	☆☆	—	☆	☆	5
Lacquemant et al.	2002	—	—	☆	☆	—	☆	☆	☆	5
Lu et al.	2006	—	☆	☆	☆	—	☆	☆	—	5
Sharma et al.	2018	☆	—	☆	☆	☆	☆	☆	—	6
Shokouhi et al.	2015	☆	☆	☆	☆	☆	☆	☆	☆	8
Sun et al.	2006	—	☆	☆	☆	☆☆	☆	☆	☆	8
Weng et al.	2010	—	☆	—	☆	☆☆	☆	☆	☆	7
Zhang et al.	2007	—	☆	☆	☆	☆	☆	☆	☆	7
Zhu et al.	2017	—	☆	—	☆	☆	☆	☆	☆	6
Zhu et al.	2009	☆	☆	☆	☆	☆	☆	☆	☆	8

Note: a study can be awarded a maximum of one star for each numbered item within the Selection and Exposure categories. A maximum of two stars can be given for Comparability. A maximum of 2 stars can be allotted in this category, one for age, the other for other controlled factors (gender, BMI, WHR, and so on).

**Table 3:** Summary of meta-analysis of association between PGC-1*α*rs8192678 polymorphism and T2DM.

Genetic model	Pooled OR (95% CI)	*Z* value	*P* value	Study number	Cases	Controls	*I* ^2^ (%)	*P*_heterogeneity_
Allelic genetic model	1.24 (1.13-1.35)	4.76	<0.05	20	8038	8144	66.4	<0.05
East Asian subgroup	1.15 (1.02-1.29)	2.34	<0.05	10	3972	4618	67.9	<0.05
Caucasian subgroup	1.28 (1.09-1.49)	3.07	<0.05	7	2927	2246	65.4	<0.05
Indian subgroup	1.35 (1.12-1.62)	3.13	<0.05	2	905	1042	39.8	0.19
African subgroup	1.84 (0.90-3.74)	1.68	0.09	1	234	238	58.1	0.12
Dominant genetic model	1.27 (1.14-1.42)	4.27	<0.05	20	8038	8144	57.5	<0.05
East Asian subgroup	1.15 (1.00-1.32)	1.91	0.06	10	3972	4618	51.1	<0.05
Caucasian subgroup	1.37 (1.10-1.71)	2.84	<0.05	7	2927	2246	64.8	<0.05
Indian subgroup	1.54 (1.12-2.11)	2.68	<0.05	2	905	1042	61.2	0.08
African subgroup	1.28 (0.77-2.13)	0.97	0.33	1	234	238	0	0.71
Recessive genetic model	1.24 (1.14-1.36)	4.95	<0.05	20	8038	8144	45.5	<0.05
East Asian subgroup	1.17 (1.04-1.31)	2.63	<0.05	10	3972	4618	64.9	<0.05
Caucasian subgroup	1.31 (1.12-1.53)	3.45	<0.05	7	2927	2246	0	0.52
Indian subgroup	1.35 (1.02-1.78)	2.08	<0.05	2	905	1042	0	0.88
African subgroup	18.85 (2.55-139.61)	2.87	<0.05	1	234	238	0	0.34
Homozygous genetic model	1.40 (1.20-1.64)	4.34	<0.05	20	8038	8144	50	<0.05
East Asian subgroup	1.31 (1.04-1.64)	2.3	<0.05	10	3972	4618	64.8	<0.05
Caucasian subgroup	1.47 (1.21-1.79)	3.84	<0.05	7	2927	2246	4.9	0.39
Indian subgroup	1.59 (1.18-2.14)	3.06	<0.05	2	905	1042	0	0.54
African subgroup	13.63 (1.71-108.58)	2.47	<0.05	1	234	238	0	0.36
Heterozygous genetic model	1.20 (1.06-1.35)	2.87	0.004	20	8038	8144	59.5	<0.05
East Asian subgroup	1.08 (0.94-1.24)	1.07	0.29	10	3972	4618	45.4	0.06
Caucasian subgroup	1.32 (1.05-1.66)	2.37	0.02	7	2927	2246	63.9	<0.05
Indian subgroup	1.52 (1.08-2.13)	2.40	0.02	2	905	1042	62.9	0.07
African subgroup	0.63 (0.20-2.07)	0.76	0.45	1	234	238	68.3	0.08

A, mutant type; genetic model: allelic (A vs. G), dominant (AG + AA vs. GG), recessive (AA vs. AG + GG), homozygous (AA vs. GG), and heterozygous (AG vs. GG).

## Abbreviations

T2DM: Type 2 diabetes mellitus
PGC-1*α*: Peroxisome proliferator-activated receptor *γ* coactivator-1*α*
NOS: Newcastle-Ottawa Scale
HWE: Hardy-Weinberg equilibrium.
